# Effects of a Four-Day Mindfulness Intervention on Teachers’ Stress and Affect: A Pilot Study in Eastern China

**DOI:** 10.3389/fpsyg.2020.01298

**Published:** 2020-06-30

**Authors:** Xiaolan Song, Ming Zheng, Huiwen Zhao, Tianqi Yang, Xingcheng Ge, Hongmei Li, Ting Lou

**Affiliations:** ^1^Laboratory of Mindfulness, Department of Psychology, College of Teacher Education, Zhejiang Normal University, Jinhua, China; ^2^MindUp Mindfulness Center, Hangzhou, China

**Keywords:** mindfulness, intensive mindfulness training, teachers’ stress, mental health, positive affect, negative affect

## Abstract

Stress is becoming increasingly prevalent among teacher groups, and this is problematic for education. Mindfulness training (MT) is a well-supported way to help various populations cope with and reduce stress. In this study, a 4-day intensive MT program that aimed to increase teachers’ emotional health was developed and implemented into the existing post-service education for teachers in eastern China. A total of 161 teachers voluntarily enrolled in the course and were assigned to either the mindfulness group or the waitlist group. Participants completed measures of mindfulness, positive affect, negative affect, and perceived stress before and after the program. The results showed that MT had statistically significant positive effects on mindfulness, negative affect, and stress. The present findings indicate that a 4-day intensive MT program is a promising way to decrease teachers’ stress and improve their emotional health. The practical meaning of the short-term intensive MT program for teachers is discussed. It is easier for teachers to enroll such a short-term training program, as it may have higher acceptance and feasibility than an 8-week training program in some areas.

**Clinical Trial Registration:** www.ClinicalTrials.gov, identifier ChiCTR2000029653.

## Introduction

Teaching is a stressful occupation (Roeser et al., 2013). Teachers not only experience pressures similar to other professions (workload, reputation pressure, etc.), but there are also profession-specific pressures, such as having to fulfill multiple roles (such as caregiver, counselor, disciplinarian, information provider, etc.) and balancing educational responsibilities against the stress and pressure from students, parents, and outside sources. Between 25 and 30% of teachers rate their jobs as either very or extremely stressful (Kyriacou, 2001; Unterbrink et al., 2007). According to the report on national mental health development in China (2017–2018), the overall mental health of teachers decreases year by year. Eastern China has a fast-developing economy that is also driving the development of education, and the high competition brought on by this development increases pressure on teachers. The main psychological problems of teachers are anxiety and depression (Fu et al., 2019). Studies show that the overall mental health condition of Chinese teachers is much worse than normal and that some teachers have a diagnosable emotional disorder (Li, 2006; Hu et al., 2010; Yi et al., 2014).

There are several factors associated with high levels of stress among teachers. According to reports from teachers, teaching involves considerable pressure for several important reasons, such as lack of support and excessively burdensome responsibilities (Jennings et al., 2017). Pithers and Soden (1998) examined the occupational stress, strain, and personal coping resources of teachers and found that role overload appeared to be a strong source of occupational stress. Zembylas and Schutz (2009) proposed that teaching stress is primarily due to the inherent social-emotional demands that entail working with 30 or more children or adolescents at once. The uncertain and emotionally taxing teaching requirement can significantly lower their well-being and instructional practice for those teachers who have not developed the mental habits to effectively manage relevant resources and demands (Roeser et al., 2012). This can become a vicious circle: a difficult situation causes failure, which lowers the teacher’s self-esteem and confidence, resulting in higher stress that, in turn, makes the situation more difficult. Increasing levels of stress in teachers also negatively affects their health. Negative emotions resulting from chronic high levels of stress may impair the cognitive functioning and well-being of teachers and lower the quality of their instruction (Emmer and Stough, 2001). Other studies also found that stress and negative affect can lead to work absenteeism and a diminished capacity to engage and effectively teach students (Roeser et al., 2012). In sum, stress and negative emotions can undermine a teacher’s health and well-being, which, in turn, will negatively impact the students (Roeser et al., 2013).

One method for reducing stress and improving well-being practicing mindfulness (Jennings et al., 2017). There is evidence that mindfulness-based interventions (MBIs) can reduce stress among various populations (Shapiro et al., 2005; Chiesa and Serretti, 2009). Empirical support of a beneficial effect exists for multiple types of MBIs, including the 8-week mindfulness-based stress reduction (MBSR) program (Kabat-Zinn, 2003), mindfulness-based cognitive therapy (MBCT; Teasdale et al., 2000), and mindfulness-based relationship enhancement (MBRE; Carson et al., 2004). Evidence shows that mindfulness training (MT) strongly helps adults reduce stress and improve emotions by heightening their awareness of the present moment without judgment (Grossman et al., 2004; Carmody and Baer, 2008).

There is also increasing evidence that MBIs could help teachers cope with occupational stress and improve their mind-body health (Gold et al., 2010; Emerson et al., 2017; Jennings et al., 2017). Flook et al. (2013) conducted a pilot study to assess mindfulness effects in teachers and found significant reductions in psychological symptoms and burnout after the intervention. Mindfulness-based training can effectively reduce stress and burnout as well as symptoms of anxiety and depression at follow-up (Roeser et al., 2013); it also shows promise in improving emotional regulation among teachers (Emerson et al., 2017). Cultivating Awareness and Resilience in Education (CARE for Teachers), a mindfulness-based professional development (PD) program designed to promote teachers’ social and emotional competence and to improve the quality of classroom interactions, has proved to be an effective PD tool both for promoting teachers’ social and emotional competence and increasing the quality of their classroom interactions (Jennings et al., 2017).

Despite the potential benefits of such interventions, for people who suffer from stress, many of the MBIs offered to teachers are long-term interventions that require a considerable investment of time, energy, and money (Zeidan et al., 2010a), especially for the people who suffer from stress. Usually, MBIs are conducted as 8- to 10-week courses in groups of up to 30 participants who meet weekly for 2–2.5 h (Baer, 2003). Consequently, due to the long and sustained commitment required, individuals may have little chance to enroll in an 8- or 10-week MBI course (2–3 h per week). Therefore, some researchers investigated whether it would be equally effective to use short-term MT. Previous research, such as that of Tang et al. (2007), reported that 5 days of Integrative Body-Mind Training (20 min/day) improved mood and cognitive processes. Furthermore, while a 10-day intensive MT (10 h/day) may reduce psychological distress (Ostafin et al., 2006), a 3-day mindfulness meditation (20 min/day) was also verified effective in reducing pain rating and anxiety scores and was also effective at increasing mindfulness skills (Zeidan et al., 2010a). A similar 3-day training (1-h total) also indicated that the brief meditation intervention was more effective at reducing negative mood, depression, fatigue, confusion, and heart rate when compared to the sham and control groups (Zeidan et al., 2010b). However, few studies have focused on the effects of short-term MT on reducing stress in teachers and none of systematic MTs designed for teachers to solve the psychological problems.

In an effort to contribute to this research space, we designed a 4-day intensive MT workshop for teachers who do not have enough time or financial support to enroll in an 8-week mindfulness-based course. Teachers, like other professionals, need to stay informed about new knowledge and technologies. They are required to take a PD course. Usually, PD courses supported by the government are the main forms of post-service education not only in China but also in many other countries. The forms of PD courses include workshops, conferences, study groups, professional, peer coaching days, etc. for days, weeks, or months (Garet et al., 2001). Professional development workshops lasting several days are regarded effective for teachers and are applied to different subjects (Yang, 2011). However, neither teacher education nor PD programs prepare teachers for psychological need (Jennings and Greenberg, 2009). Therefore, our 4-day intensive MT implemented into PD program could benefit teachers who are suffering from stress-related issues. Besides, this program could be the first step for teachers to learn how to cope with stress through mindfulness more easily and prepare them to participate in long-term MT such as MBCT or MBSR.

The aim of this study was to test whether a 4-day intensive MT can reduce teachers’ stress, reduce negative emotions, and increase positive emotions a day before and after intervention. Consequently, our hypotheses were that teachers who participated in 4-day intensive MT would show higher mindfulness traits, less stress, less negative affect, and more positive affect than at baseline and when compared with wait-listed teachers.

## Method

The research procedure was in accordance with the ethical principles of the 1964 Declaration of Helsinki (World Medical Organization). The Human Research Ethics Committee approved the research procedure. Informed written consent was obtained from all individual participants included in the study.

### Study Design

**FIGURE 1:**
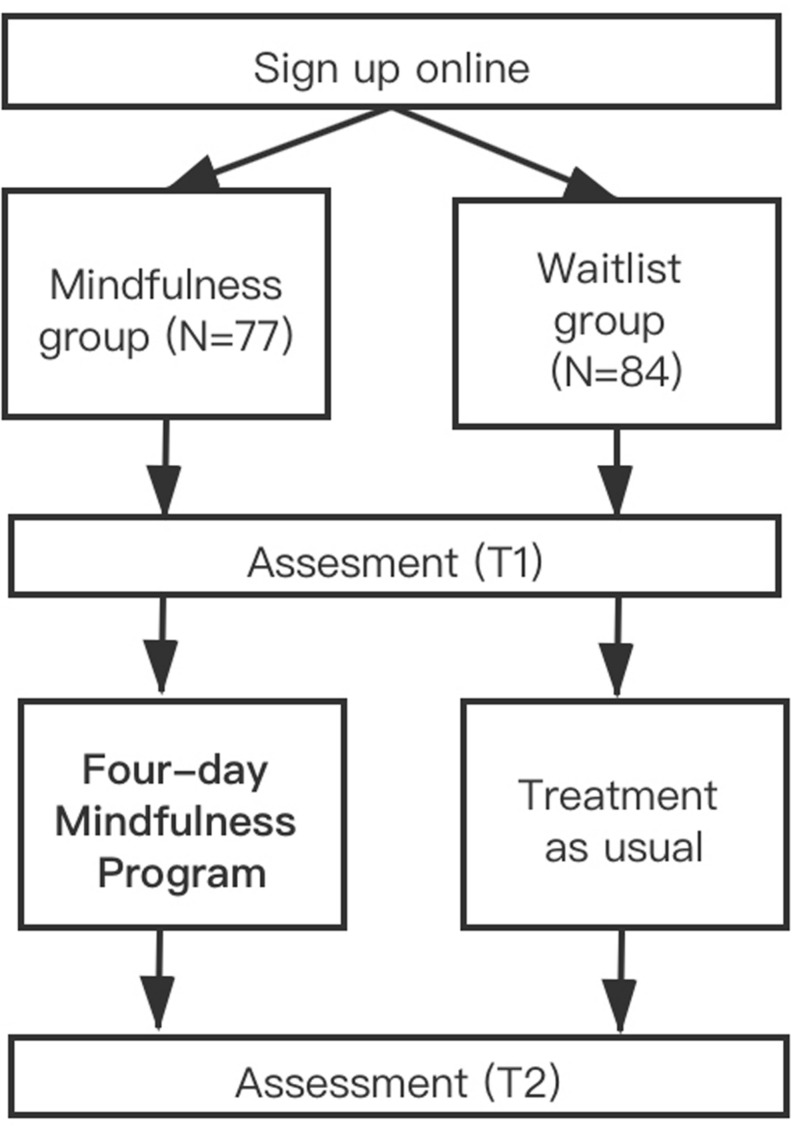
Flowchart of the process.

Teacher recruitment took place in the Spring of 2018 in Zhejiang province, China. This 4-day intensive mindfulness workshop was part of the Teacher Training Project in Zhejiang province, which received financial support from the government. Participating teachers received the training for free and could acquire credits for their PD. All teachers voluntarily participated in this project and signed up through the online platform of the Education Department of Zhejiang. Teachers were assigned to the April or July course according to their chosen time schedule. The group taking the April course was labeled as the mindfulness-training group and the July course as the waitlist group. Participants completed a baseline assessment (T1) 1 day before commencing the April course and post-assessment (T2) 1 day after completing the April course using the online questionnaire software SO JUMP. Participants were assigned a course number when they signed up for the program online, and all questionnaires were anonymously completed using the assigned numbers. They were instructed to respond to the questionnaire items according to their situation using the scales. The waitlist group completed the assessments at the same time as the mindfulness-training group, and the instructions for both groups were the same (see Figure 1).

### Participants

Participants signed into the program via the online registration system of the Department of Education of Zhejiang Province. The final mindfulness group included 77 public school teachers (67 women), and the final waitlist group included 84 public school teachers (70 women). Most of (153 teachers) the participants were from primary schools, middle schools, and high schools. Only three teachers were from a college or a university. The age of the participants ranged from 24 to 55 years (*M* = 38.50 years, SD = 6.78, median = 38). The teaching experience ranged from 2 to 38 years (*M* = 16.81 years, SD = 8.14, median = 16). Of the teachers, 68.83% of the mindfulness group and 60.71% of the waitlist group were from middle school and high school, which was the majority (see details in Table 1).

**TABLE 1:** Demographic information of the participating teachers (*N* = 161).

	Mindfulness group (*n* = 77)	Waitlist (*n* = 84)
Preschool	4	1
Primary school	18	31
Middle school	18	24
High school	35	27
Universities or colleges	2	1
Sex (% female)	87%	83.33%
Age in years, mean (range)	38.42 (25–55)	38.57 (24–55)
Years of teaching experience, mean (range)	16.81 (2–34)	16.82 (3–38)

### Intervention Procedures

We adapted the 4-day Intensive Mindfulness Training Program from MBSR (Kabat-Zinn, 2003). The practices in the course included the classical practice of MBSR like sitting meditation, mindful eating, and body scanning, and so on. The training was conducted by two qualified mindfulness instructors who had received MBSR and MBCT teacher training and had more than 1000 h of mindfulness meditation experience. Participants checked in one night before the first day of the class and stayed overnight during the course. The course lasted 8 h from 9 am to 5 pm with 2 h midday rest on each day. All participants completed the intervention together in a hall of university. On the first day, after a brief introduction of mindfulness, the participants started mindfulness practice under the guidance of the teacher, and, at the end of the first day, they were given a short lecture about “what we do when we practice mindfulness,” which aimed to make the participants understand the mindfulness practice. The course was taught in accordance with the basic rule of MBI; that is, the teaching was tightly based on the participants’ practices. This was followed by a question-and-answer session, which intended to deepen the participants’ experience during practices. It is worth mentioning that the third day entirely involved silent retreat, and the discussion was conducted only at the end of the day (see Supplementary Table 1 for the course schedule).

### Measures

#### Mindfulness

Mindfulness was assessed using the Chinese version of the Mindful Attention Awareness Scale (MAAS; Brown and Ryan, 2003; Carlson and Brown, 2005; Chen et al., 2012). This 15-item scale was designed to assess a core characteristic of mindfulness, namely, a receptive state of mind in which attention, informed by a sensitive awareness of what is occurring in the present, simply observes what is taking place (e.g., “I could be experiencing some emotion and not be conscious of it until sometime later.”). Items are rated on a 6-point Likert scale (1 = Almost always to 6 = Almost never). The scales were statistically reliable (Cronbach’s alpha = 0.87).

#### Stress

Stress was assessed using the Chinese Perceived Stress Scale (CPSS: Chinese Perceived Stress Scale: Cohen et al., 1997; Yang and Huang, 2003). This 14-item scale is the most widely used psychological instrument for measuring the perception of stress (e.g., “In the last month, how often have you been upset because of something that happened unexpectedly?”). Items are rated on a 5-point Likert scale (1 = Never to 5 = Very often). Items 4, 5, 6, 7, 9, 10, and 13 are the positively stated items, which are reverse scored. Higher scores indicate higher perceived stress. The scales were statistically reliable (Cronbach’s alpha = 0.89).

#### Affect

Positive affect and negative affect were measured by the Chinese Positive and Negative Affect Schedule (Watson et al., 1988; Huang et al., 2003). The scale consists of 20 different words to describe positive (10 items) or negative (10 items) feelings and emotions and has proven reliable and valid (e.g., “afraid, active, alert, scared”). Items are rated on a 5-point Likert scale (1 = Very slightly or not at all to 5 = extremely). Each sub-scale was statistically reliable (Positive affect: Cronbach’s alphas = 0.89; negative affect: Cronbach’s alpha = 0.89).

### Data Analysis

Descriptive statistics and correlation analysis were conducted to show the baseline situation of participants. Repeated-measure ANOVAs were conducted appropriate to the 2 × 2 factorial design.

## Results

### Preliminary Analyses and Demographic Information

Participants reported their responses voluntarily. After data cleaning, the distribution, outliers, and homogeneity of data were examined. All the Cronbach alphas were more than 0.69. In case of the common method variance, we conducted the Harman’s single factor test; the total variance for one factor was 32.42%, which is below 40% (Podsakoff et al., 2003).

Table 1 shows the demographic information of the two groups, including the detailed classification of participants, gender, age, and years of teaching. There were no significant differences between the two groups in terms of gender, χ^2^ = 0.43, *p* = 0.51, age, *t*(159) = −0.40, *p* = 0.69 and teaching experience, *t*(159) = −0.01, *p* = 0.99. Results also revealed no significant difference between the two groups in years of teaching experience, *t*(159) = −0.01, *p* = 0.99 (see Table 1).

Table 2 shows descriptive statistics and correlations between outcomes at baseline. The Pearson’s correlation test revealed that dispositional mindfulness of participants was positively associated with positive affect, *r* = 0.43^∗∗∗^, 95%CI [0.30, 0.55] and negatively related to negative affect, *r* = −0.53^∗∗∗^, 95%CI [−0.63. −0.40] and stress, *r* = −0.59^∗∗∗^, 95%CI [0–0.68, −0.48] at baseline (see Table 2).

**TABLE 2:** Descriptive statistics and correlations between outcomes at baseline (*N* = 161).

	*M* ± SD	Cronbach’s α	MAAS^a^	PA^b^	NA^c^	STRESS
MAAS	3.94 ± 0.70	0.87	–			
PA	3.11 ± 0.55	0.89	0.43*** [0.30, 0.55]^d^	–		
NA	2.20 ± 0.56	0.89	−0.53*** [−0.63, −0.40]	−0.42*** [−0.54, −0.28]	–	
STRESS	2.65 ± 0.52	0.89	−0.59*** [−0.68, −0.48]	−0.65*** [−0.73, −0.55]	0.73* [0.65, 0.80]	–

^*a*^MAAS, Mindfulness level; ^*b*^PA, Positive affect of PANAS; ^*c*^NA, Negative affect of PANAS; ^*d*^ Pearson’s *r*; [95% Confidence Interval]. *p < 0.05, **p < 0.01, ***p < 0.001.

### Intervention Effects on Teachers’ Mindfulness, Affect, and Stress

**FIGURE 2:**
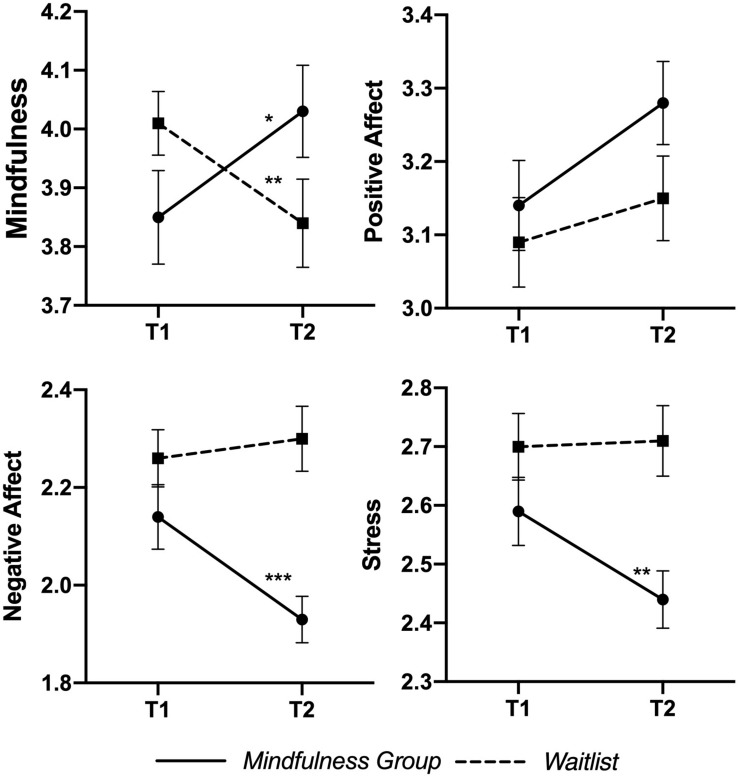
Changes in outcomes for the mindfulness and waitlist groups. T1 = Baseline measurement; T2 = post-program measurement; Error bar = ±1SEM.

Table 3 presents the impacts of the training on the four variables of interest: mindfulness, positive affect, negative affect, and stress. At baseline, there were no significant differences in any measurement between the two groups. Repeated ANOVA results revealed a significant intervention (mindfulness group, waitlist) × assessment time (T1, T2) interaction on mindfulness, *F*(1,159) = 13.36^∗∗∗^, η*^2^* = 0.077, negative affect, *F*(1,159) = 7.06^∗∗^, η*^2^* = 0.042, and stress, *F*(1,159) = 4.22^∗^, η*^2^* = 0.025. There was no significant interaction on positive affect, *F*(1,159) = 11.33, η*^2^* = 0.008. The two groups had different directions of changes between the two assessments; teachers in the mindfulness group had a greater improvement in mindfulness and a greater decrease in negative affect and stress (see Table 3 and Figure 2).

**TABLE 3:** Results of the ANOVA of outcome measures by study condition (mindfulness training or waitlist) (*N* = 161).

Measure^a^	Time^b^	Mindfulness group (*n* = 77) *M* ± SD	Waitlist (*n* = 84) *M* ± SD	*F*	η*^2^*
Mindfulness	T1	3.85 ± 0.70	4.01 ± 0.50		
	T2	4.03 ± 0.69	3.84 ± 0.69	13.36***	0.077
PA^c^	T1	3.14 ± 0.54	3.09 ± 0.56		
	T2	3.28 ± 0.50	3.15 ± 0.53	1.33	0.008
NA^d^	T1	2.14 ± 0.58	2.26 ± 0.54		
	T2	1.93 ± 0.42	2.30 ± 0.61	7.06**	0.042
STRESS	T1	2.59 ± 0.51	2.70 ± 0.52		
	T2	2.44 ± 0.43	2.71 ± 0.55	4.22*	0.025

^*a*^self-report outcome. ^*b*^T1 = baseline; T2 = post-program. ^*c*^PA, positive affect of PANAS. ^*d*^NA, negative affect of PANAS. *p < 0.05, **p < 0.01, ***p < 0.001. η^2^ = effect size.

Simple main effects showed that, after the four-day intensive MT, all the variables of the mindfulness group changed significantly in the expected direction: the teachers reported greater mindfulness levels at post-program than at baseline, *F*(1, 76) = 5.33, *p* = 0.02, η*^2^* = 0.032. The teachers in the waitlist group reported lower mindfulness levels at post-program, *F*(1, 83) = 8.25, *p* = 0.005, η*^2^* = 0.049. Participation in the MT led to a statistically significant decrease in negative affect, *F*(1, 76) = 10.15, *p* = 0.001, η*^2^* = 0.060, and stress, *F*(1, 76) = 7.16, *p* = 0.008, η*^2^* = 0.043. Although there is no significant interaction effect in the positive affect, a paired sample *t*-test showed that positive affect increased significantly in the mindfulness group, *t*(76) = −2.75, *p* = 0.004, *d* = −0.31.

## Discussion

The goal of this study was to investigate whether participation in a short, intensive MT could improve well-being and reduce stress in teachers and whether such a workshop in the system of continuing education was acceptable and feasible. Overall, our results suggested that our pioneering mindfulness-training workshop could increase teachers’ mindfulness levels and, in turn, improve their emotional health, which was manifested as lower negative affect and stress. Our study promotes MT in teacher groups and shows that such an intensive 4-day project adding MT into the teachers’ continuing education system can be of great value.

The correlation pattern among mindfulness, negative/positive affect and stress at baseline was in accordance with former studies that revealed that higher dispositional mindfulness is associated with lower negative emotion (Schutte and Malouff, 2011) and higher positive emotion and self-regulated behavior (Brown and Ryan, 2003). Furthermore, the decreased scores of negative affect and perceived stress manifested the effects of such mind-body practices on teachers’ emotional health. These results are consistent with the findings of other studies on short MT (Tang et al., 2007; Harnett et al., 2010; Josefsson et al., 2014) as well as an 8-weeks  MT  for teachers (Winzelberg and Luskin, 1999; Gold et al., 2010; Benn et al., 2012; Roeser et al., 2013).

These results suggest that bringing MT to the teacher population can help them cope with stress effectively and improve their emotional health. Considering the negative impact of teacher’s stress and burnout on education quality (Hoglund et al., 2015), the positive effect of MT on the teachers could be transferred to their class and students. CARE, a well-developed mindfulness-based project for teachers’ social and emotional competence (Jennings et al., 2017), indicated that bringing mindfulness to teachers was valuable for not only teachers themselves but also students. Whether the effects of our 4-day mindfulness intervention could be transferred to students should be investigated in the future.

It is worth mentioning that the effects of MT were not the same on negative and positive affect, although positive affect tended to improve after training. Former studies showed different results about the effect of the mindfulness intervention on positive emotion. Schroevers and Brandsma (2010) found that MBCT can improve positive emotion, while other studies did not find any significant impact (Liu et al., 2013; Xu et al., 2015). One possible explanation is the design of the course. The practices during the course were mainly adapted from MBSR and MBCT, which were more focused on developing a new relation to one’s negative experience (Ostafin et al., 2015; Xu et al., 2015). Introducing compassion and appreciation practice would improve the positive affect more significantly (Galla, 2016). Another reason may be due to the healthy sample in our study; the relatively high positive emotional baseline makes the change not so obvious. In fact, the participants from Schroevers and Brandsma’s study had mild to moderate psychological problems, and the participants in Liu’s study were healthy.

An interesting finding in this study is that the mindfulness levels of the waitlist group decreased between T1 and T2. The value of mindfulness in the waitlist group at T2 was close to the value of the mindfulness group at T1, while the value of mindfulness in the mindfulness group at T2 was close to the value of the waitlist group at T1. It is important to address whether the mindfulness level oscillated by itself during that time. No studies have discussed that issue before, but we believed that the mindfulness level improved after intervention based on the following reasons. First, considering the matched occupation and motivation between the two groups, no reason can explain why mindfulness would oscillate in different directions for the two groups across the same period. Furthermore, the significant increase revealed by repeated ANOVA avoided the problems brought on by the baseline differences and repeated assessments. Second, from the perspective of training, the increase was reasonable since the intervention was designed to guide participants to start paying attention unjudgementally to their present-moment experiences, which is consistent with the main contents of MAAS. Third, the other outcomes of mental health had changes similar to the mindfulness values. The additional mediation analysis (see Supplementary Materials 2) showed that the changes in other outcomes are mediated by the improvement of mindfulness levels. These results are in accordance with the theory about mindfulness and mental health and could not be explained by natural oscillation. Thus, we still conclude that the improvement of mindfulness was due to the intervention. However, it is important to note that a measure to assess mindfulness is still under development (Sauer et al., 2012; Duan, 2014).

We believed that the positive effects of this workshop were mainly due to the detailed design. Every effort was made to help teachers in acquiring mindfulness skills. This 4-day intensive mindfulness-training workshop was based on MBSR and introduced mindfulness step by step. First, the participants would gain direct experience with mindfulness through practice; then, a lecture about the science of mindfulness explained the basic principles of MT and clarified the intention of practice, which could introduce them to what mindfulness is and what MT involves. Unlike the clinical population in common MBSR programs, the teachers enrolled in the continuing education system had no serious personal distress. Teaching mindfulness to such a group and making the participant devoted to practice is not easy, and motivation stimulation can be quite important. By beginning with practice, we set the base tone of the course and the following lecture in the first afternoon, resolving any suspicions the participants might hold by motivating them. Another important setting of this program is the one-day silent retreat during the third day. Some studies showed that days of an intensive mindfulness retreat can improve executive function, which is critical to emotion regulation (Chambers et al., 2008; Sahdra et al., 2011) and a one-day silent retreat is also an essential part of MBSR and MBCT (Jha et al., 2010). Additionally, mindfulness instructors are required to attend more than 5 days of silent retreat every year in many mindfulness teaching systems to maintain their qualification (Fosarelli, 2010). Therefore, we believed that an immersive experience of mindfulness practice is quite important. In the group sharing, participants did report that the silent retreat gave them a deeper understanding of mindfulness.

The most valuable aspect of this work is that it was the first time that a mindfulness-based course was added to the existing continuing PD system in China. As a pilot study, there are several things to note about this 4-day intensive MT for teachers also bring several things to note. First, the imbalance between teachers’ high professional exhaustion (Wang and Xu, 2004) and the scarcity of psychological services provided to teachers (Wang et al., 2010) is serious not only in China but also in many other countries. The skills of paying attention to the present moment cultivated by MT could be practiced by oneself, which makes the MBIs particularly meaningful to teachers. Once they have understood and mastered the method of MT, they can continue practice by themselves in their daily lives, meaning that they attain an effective mental health maintenance tool. Segal et al. (2002) argued that regular mindfulness practice strengthens one’s ability to cope with stress, and this argument was also supported by the finding that formal meditation practiced during MBSR course is positively associated with improvements in symptoms and wellbeing (Carmody and Baer, 2008). Second, teachers are required to receive regular PD courses under the support of government and those courses usually last several days. Unlike a long-term mindfulness course like MBSR, the value of a short-term mindfulness course that is this program can be incorporated into the existing PD education system. This 4-day intensive MT resembles other continuing courses, whereby teachers have the opportunity to enroll at no extra time and cost.

It is worth mentioning that value of this course, which could not be presented in the quantitative data, is mainly reflected in the experiences sharing by the participants. One of the insights that the participants reported the most was the great transformative power of focusing attention on the present moment, especially the body sensation. This was a skillful method to keep being with the sensation instead of automated reaction when emotional thoughts come. That is, MT brings a practical way to cope with affects and feeling and cultivate awareness of the present moments in a non-reactive and non-judgmental manner which facilitates emotion regulation, stress reduction (Roeser et al., 2012). This stepping out from the thought-emotion vortex was named “decentering,” which is the key mechanism of mindfulness meditation transforming emotional disorders and could be cultivated by persistence mindfulness practice (Segal et al., 2019). The present study indicated that the benefits could be perceived by teachers in just four-day training. Second, intensive and relatively long-time mindfulness practice provided them opportunity to taste the inner peace behind the mind which was usually ignored in busy lives. For example, in the discussion session of the 1-day silent retreat (the third day), several teachers shared their experience of mindful lunch. The experience of mindful eating gave them insight about the importance of connection with the direct experience. They sincerely talked about the feelings when pay attention to the present moment and how the smell, the taste, and the texture of a grain of rice made them touched. Third, most of teachers were willing to recommend the course to their colleagues, which was also common in mindfulness projects in other countries (Mindful schools, 2010). We believe that the deep feelings and insights they obtained through the course could give them a preliminary mindfulness experience and help them to be prepared for longer trainings.

There are also a number of potential challenges in implementing mindfulness into existing teacher PD. First, the benefits of such a short-term MT program for teachers need to be confirmed, while for managing teachers’ stress, there is only limited evidence. Mindfulness-based interventions was far from being accepted in teacher mental health maintenance. Second, promoting the program is also difficult. Usually, teachers could only enroll in the courses that are in accordance with their teaching projects rather than a new psychological course. Additionally, most of the psychological course was only provided to school psychologists. Attending such a program needs to be supported by school leaders, and this is also true for other countries. Having a supportive head teacher and senior leadership team is very important for planting mindfulness in schools (Wilde et al., 2019). For the present study, we classified this course in the category of “teachers’ moral education” so teachers who teach any projects could sign up. Third, entirely devoting into mindfulness practice for MBI is quite important, which requires a quiet and clean environment. Therefore, we took several ways to make the proper arrangements, for example, choosing quiet and spacious hall, reminding teachers to arrange work in advance, taking care of their mobile phones to avoid interruptions. This was all part of the entire program.

There are four limitations to this study. First, it is important to note that the effect sizes in this study ranged from small to medium. The relatively small effect size of mindfulness interventions has received attention for a long time and is a common phenomenon in MBI studies. Grossman et al.’s (2004) review of MBI studies found that both controlled and uncontrolled studies showed similar effect sizes of approximately medium effect and that the moderated effect size in healthy groups is more common. A systematic review conducted in adolescents and young adults found that MBSR had only moderate effects in reducing depressive symptoms at the end of the intervention. The small effect size may be due mostly to the short intervention (Goldberg et al., 2017). Cavanagh et al. (2018) reported small to medium effect sizes in all measured domains in their study, which was a brief MBI in a non-clinical population. Another study failed to distinguish effects in the mindfulness group compared to an active control and waitlist group after an online short-term MBI (Josefsson et al., 2014). The small effect sizes in these studies are likely due to the short and shallow nature of the interventions. In the same way, most studies on silent retreat, which required longer practice and deeper devotion, showed medium to large effect sizes (Khoury et al., 2017). According to these results, future studies need to confirm the effectiveness of short-term mindfulness interventions. Second, it is possible that the decline of stress and negative emotion among the participants was partly due to their absence from their jobs. One study reported improvements in psychological outcomes in the meditation group compared to a control group that took a brief leisure vacation (Wong and Oi, 2011). However, future studies should still consider adopting an active control. Third, due to the work arrangements of the participants, it is difficult to ensure a standardized randomization, although there were no significant differences between the two groups at baseline. Fourth, because the participants were recruited from different schools and different areas of Zhejiang Province, we could not collect follow-up data. Future studies should focus on the long-term benefits of such a short-term mindfulness workshop as well as examine whether the participants continue their practices.

## Summary

Our results indicated that a 4-day intensive MT can improve teachers’ mindfulness and reduce their stress and negative affect, which is quite important for those teachers who are suffering from occupational stress and other mental distress. We do not suggest that brief MT is as effective as long-term developed training, such as MBSR or MBCT. However, when time or financial support is limited, a short-term training program may have higher acceptance and feasibility than an 8-week training program in some areas.

## Data Availability Statement

The datasets generated for this study are available on request to the corresponding author.

## Ethics Statement

The studies involving human participants were reviewed and approved by Institutional Research Committee of Zhejiang Normal University. The patients/participants provided their written informed consent to participate in this study.

## Author Contributions

XS and MZ conceived, designed, and executed the study, analyzed the data, and wrote the manuscript. HZ, TY, and XG collected the data. HZ analyzed the data and edited the final version of the manuscript. XS, TL, and HL designed the 4-day intensive mindfulness training course. All authors have read and approved the final version of the manuscript for submission.

## Conflict of Interest

TL was employed by commonwealth organization “MindUp Mindfulness Center”. The remaining authors declare that the research was conducted in the absence of any commercial or financial relationships that could be construed as a potential conflict of interest.

## References

[B1] Baer R. A. (2003). Mindfulness training as a clinical intervention: a conceptual and empirical review. *Psychol. Sci. Prac.* 10 125–143. 10.1093/clipsy/bpg015

[B2] Benn R. Akiva T. Arel S. Roeser R. W. (2012). Mindfulness training effects for parents and educators of children with special needs. *Dev. Psychol.* 48 1476–1487. 10.1037/a0027537 22409766

[B3] Brown K. W. Ryan R. M. (2003). The benefits of being present: mindfulness and its role in psychological well-being. *J. Pers. Soc. Psychol.* 84 822–848. 10.1037/0022-3514.84.4.822 12703651

[B4] Carlson L. E. Brown K. W. (2005). Validation of the Mindful Attention Awareness Scale in a cancer population. *J. Psychosom. Res.* 58 29–33. 10.1016/j.jpsychores.2004.04.366 15771867

[B5] Carmody J. Baer R. A. (2008). Relationships between mindfulness practice and levels of mindfulness, medical and psychological symptoms and well-being in a mindfulness-based stress reduction program. *J. Behav. Med.* 31 23–33. 10.1016/S0005-7894(04)80028-5 17899351

[B6] Carson J. W. Carson K. M. Gil K. M. Baucom D. H. (2004). Mindfulness-based relationship enhancement. *Behav. Ther.* 35 471–494. 10.1016/S0005-7894(04)80028-5

[B7] Cavanagh K. Churchard A. O’Hanlon P. Mundy T. Strauss C. (2018). A randomised controlled trial of a brief online mindfulness-based intervention in a non-clinical population: replication and extension. *Mindfulness* 9 1–15. 10.1007/s12671-017-0856-1 30100934 PMC6061247

[B8] Chambers R. Lo B. C. Y. Allen N. B. (2008). The impact of intensive mindfulness training on attentional control, cognitive style, and affect. *Cognit. Ther. Res.* 32 303–322. 10.1007/s10608-007-9119-0

[B9] Chen S. Cui H. Zhou R. Jia Y. (2012). Revision of Mindful Attention Awareness Scale(MAAS). *Chin. J. Clin. Psychol.* 20 148–151.

[B10] Chiesa A. Serretti A. (2009). Mindfulness-based stress reduction for stress management in healthy people: a review and meta-analysis. *J. Altern. Complement. Med.* 15 593–600. 10.1089/acm.2008.0495 19432513

[B11] Cohen S. Kessler R. C. Gordon L. U. (1997). Measuring stress? a guide for health and social scientists. *Oxford Univ. Press Demand* 41:186 10.1016/0022-3999(96)00066-9

[B12] Duan W. (2014). Disagreements of studies on mindfulness: conceptualization and measurements. *Adv. Psychol. Sci.* 22:1616 10.3724/SP.J.1042.2014.01616

[B13] Emerson L. Leyland A. Hudson K. Rowse G. Hanley P. Hugh-Jones S. (2017). Teaching mindfulness to teachers: a systematic review and narrative synthesis. *Mindfulness* 8 1136–1149. 10.1007/s12671-017-0691-4 28989547 PMC5605579

[B14] Emmer E. T. Stough L. M. (2001). Classroom management: a critical part of educational psychology, with implications for teacher education. *Educ. Psychol.* 36 103–112. 10.1207/S15326985EP3602_5

[B15] Flook L. Goldberg S. B. Pinger L. Bonus K. Davidson R. J. (2013). Mindfulness for teachers: a pilot study to assess effects on stress, burnout, and teaching efficacy. *Mind Brain Educ.* 7 182–195. 10.1111/mbe.12026 24324528 PMC3855679

[B16] Fosarelli P. (2010). Teaching mindfulness: a practical guide for clinicians and educators. *JAMA* 306 1004–1005. 10.1001/jama.2011.1271

[B17] Fu X. Zhang K. Chen Z. (2019). *Blue Book of Mental Health: Report On National Mental Health Development in China (2017-2018).* China: Social Sciences Academic Press.

[B18] Galla B. M. (2016). Within-person changes in mindfulness and self-compassion predict enhanced emotional well-being in healthy, but stressed adolescents. *J. Adolesc.* 49 204–217. 10.1093/scan/nsv008 27107398

[B19] Garet M. Porter A. Desimone L. Birman B. Yoon K. S. (2001). What makes professional development effective? results from a national sample of teachers. *Am. Educ. RES. J.* 38 915–945. 10.3102/00028312038004915

[B20] Gold E. Smith A. Hopper I. Herne D. Tansey G. Hulland C. (2010). Mindfulness-Based Stress Reduction (MBSR) for primary school teachers. *J. Child Fam. Stud.* 19 184–189. 10.1007/s10826-009-9344-0

[B21] Goldberg S. B. Tucker R. P. Greene P. A. Davidson R. J. Wampold B. E. Kearney D. J. (2017). Mindfulness-based interventions for psychiatric disorders: a systematic review and meta-analysis. *Clin. Psychol. Rev.* 59 52–60. 10.1016/j.cpr.2017.10.011 29126747 PMC5741505

[B22] Grossman P. Niemann L. Schmidt S. Walach H. (2004). Mindfulness-based stress reduction and health benefits. A meta-analysis. *J. Psychosom. Res.* 57 35–43. 10.1016/S0022-3999(03)00573-7 15256293

[B23] Harnett P. H. Whittingham K. Puhakka E. Hodges J. Spry C. Dob R. (2010). The short-term impact of a brief group-based mindfulness therapy program on depression and life satisfaction. *Mindfulness* 1 183–188. 10.1007/s12671-010-0024-3

[B24] Hoglund W. L. G. Klingle K. E. Hosan N. E. (2015). Classroom risks and resources: teacher burnout, classroom quality and children’s adjustment in high needs elementary schools. *J. Sch. Psychol.* 53 337–357. 10.1016/j.jsp.2015.06.002 26407833

[B25] Hu W. Ma Y. Jiao L. Wang H. (2010). Investigation of mental health level of primary and secondary school teachers in Shanxi Province. *Theory Pract. Educ.* 10 59–62.

[B26] Huang L. Yang T. Ji Z. (2003). Applicability of the Positive and Negative Affect Scale in Chinese. *Chin. Ment. Health J.* 17 54–56.

[B27] Jennings P. A. Brown J. L. Frank J. L. Doyle S. Oh Y. Davis R. (2017). Impacts of the CARE for teachers program on teachers social and emotional competence and classroom interactions. *J. Edu. Psychol.* 109 1010–1028. 10.1037/edu0000187

[B28] Jennings P. A. Greenberg M. T. (2009). The prosocial classroom: teacher social and emotional competence in relation to student and classroom outcomes. *Rev. Educ. Res.* 79 491–525. 10.2307/40071173

[B29] Jha A. P. Stanley E. A. Kiyonaga A. Wong L. Gelfand L. (2010). Examining the protective effects of mindfulness training on working memory capacity and affective experience. *Emotion* 10 54–64. 10.1037/a0018438 20141302

[B30] Josefsson T. Lindwall M. Broberg A. G. (2014). The effects of a short-term mindfulness based intervention on self-reported mindfulness, decentering, executive attention, psychological health, and coping style: examining unique mindfulness effects and mediators. *Mindfulness* 5 18–35. 10.1007/s12671-012-0142-1

[B31] Kabat-Zinn J. (2003). Mindfulness-based interventions in context: past, present, and future. *Clin. Psychol. Sci. Pract.* 10 144–156. 10.1093/clipsy.bpg016

[B32] Khoury B. Knäuper B. Schlosser M. Carrière K. Chiesa A. (2017). Effectiveness of traditional meditation retreats: a systematic review and meta-analysis. *J. Psychosom. Res.* 92 16–25. 10.1016/j.jpsychores.2016.11.006 27998508

[B33] Kyriacou C. (2001). Teacher stress: directions for future research. *Edu. Rev.* 53 27–35. 10.1080/00131910120033628

[B34] Li Y. (2006). Analysis on present situation and counter measure for psychological health of middle school teachers. *J. Dalian Edu. Univ.* 3 22–24.

[B35] Liu X. Xu W. Wang Y. Liu H. (2013). Effect of mindfulness training on subjective well-being in volunteers: a six-week randomized controlled trial. *Chin. Ment. Health J.* 27 597–601.

[B36] Mindful schools (2010). Available online at: https://www.mindfulschools.org/about-mindfulness/research-on-mindfulness/ (accessed May 11, 2020).

[B37] Ostafin B. Chawla N. Bowen S. Dillworth T. M. Witkiewitz K. Marlatt G. A. (2006). Intensive mindfulness training and the reduction of psychological distress: a preliminary study. *Cognit. Behav. Pract.* 13 191–197. 10.1016/j.cbpra.2005.12.001

[B38] Ostafin B. D. Robinson M. D. Meier B. P. (2015). *Handbook of Mindfulness and Self-Regulation.* New York, NY: Springer, 10.1007/978-1-4939-2263-5

[B39] Pithers R. T. Soden R. (1998). Scottish and Australian teacher stress and strain: a comparative study. *Br. J. Edu. Psychol.* 68 269–279. 10.1111/j.2044-8279.1998.tb01289.x 9661340

[B40] Podsakoff P. M. Mackenzie S. B. Lee J.-Y. Podsakoff N. P. (2003). Common method biases in behavioral research a critical review of the literature and recommended remedies. *J. Appl. Psychol.* 88 879–903. 10.1037/0021-9010.88.5.879 14516251

[B41] Roeser R. W. Schonert-Reichl K. A. Jha A. Cullen M. Wallace L. Wilensky R. (2013). Mindfulness training and reductions in teacher stress and burnout: results from two randomized, waitlist-control field trials. *J. Edu. Psychol.* 105 787–804. 10.1037/a0032093

[B42] Roeser R. W. Skinner E. Beers J. Jennings P. A. (2012). Mindfulness training and teachers’ professional development: an emerging area of research and practice. *Child Dev. Perspect.* 6 167–173. 10.1111/j.1750-8606.2012.00238.x

[B43] Sahdra B. K. MacLean K. A. Ferrer E. Shaver P. R. Rosenberg E. L. Jacobs T. L. (2011). Enhanced response inhibition during intensive meditation training predicts improvements in self-reported adaptive socioemotional functioning. *Emotion* 11 299–312. 10.1037/a0022764 21500899

[B44] Sauer S. Walach H. Schmidt S. Hinterberger T. Lynch S. Büssing A. (2012). Assessment of mindfulness: review on state of the art. *Mindfulness* 4 3–17. 10.1007/s12671-012-0122-5

[B45] Schroevers M. J. Brandsma R. (2010). Is learning mindfulness associated with improved affect after mindfulness-based cognitive therapy? *Br. J. Psychol.* 101 95–107. 10.1348/000712609X424195 19327220

[B46] Schutte N. S. Malouff J. M. (2011). Emotional intelligence mediates the relationship between mindfulness and subjective well-being. *Pers. Individ. Differ.* 50 1116–1119. 10.1016/j.paid.2011.01.037

[B47] Segal Z. V. Anderson A. K. Gulamani T. Dinh Williams L.-A. Desormeau P. Ferguson A. (2019). Practice of therapy acquired regulatory skills and depressive relapse/recurrence prophylaxis following cognitive therapy or mindfulness based cognitive therapy. *J. Consult. Clin. Psychol.* 87 161–170. 10.1037/ccp0000351 30431297

[B48] Segal Z. V. Williams J. M. G. Teasdale J. D. (2002). *Mindfulness-Based Cognitive Therapy for Depression: A New Approach to Preventing Relapse.* New York, NY: The Guilford Press.

[B49] Shapiro S. L. Astin J. A. Bishop S. R. Cordova M. (2005). Mindfulness-based stress reduction for health care professionals: results from a randomized trial. *Int. J. Stress Manage.* 12 164–176. 10.1037/1072-5245.12.2.164

[B50] Tang Y. Y. Ma Y. Wang J. Fan Y. Feng S. Lu Q. (2007). Short-term meditation training improves attention and self-regulation. *PNAS* 104 17152–17156. 10.1073/pnas.0707678104 17940025 PMC2040428

[B51] Teasdale J. D. Segal Z. V. Williams J. M. Ridgeway V. A. Soulsby J. M. Lau M. A. (2000). Prevention of relapse/recurrence in major depression by mindfulness-based cognitive therapy. *J. Consult. Clin. Psychol.* 68 615–623. 10.1037/0022-006X.68.4.615 10965637

[B52] Unterbrink T. Hack A. Pfeifer R. Buhl-Griehaber V. Müller U. Wesche H. (2007). Burnout and effort-reward-imbalance in a sample of 949 German teachers. *Int. Arch. Occup. Environ. Health* 80 433–441. 10.1007/s00420-007-0169-0 17294239

[B53] Wang F. Xu Y. (2004). Job burnout among elementary and high school teachers:characteristics and relationship with social support. *Acta. Psychol. Sin.* 036 568–574.

[B54] Wang Z. Li X. Zhang D. (2010). A review of teachers’ mental health in the past twenty years in China. *Psychol. Sci.* 02 126–129.

[B55] Watson D. Clark L. A. Tellegen A. (1988). Development and validation of brief measures of positive and negative affect: the PANAS scales. *J. Pers. Soc. Psychol.* 54:1063. 10.1037/0022-3514.54.6.1063 3397865

[B56] Wilde S. Sonley A. Crane C. Ford T. Raja A. Robson J. (2019). Mindfulness Training in UK secondary schools: a multiple case study approach to identification of cornerstones of implementation. *Mindfulness* 10 376–389. 10.1007/s12671-018-0982-4 31186817 PMC6558285

[B57] Winzelberg A. J. Luskin F. M. (1999). The effect of a meditation training in stress levels in secondary school teachers. *Stress Med.* 15 69–77. 10.1002/(SICI)1099-1700(199904)15:23.0.CO;2-W

[B58] Wong G. Oi C. (2011). *Live to Love as a Way to Love Your Living: Cultivating Compassion by Loving-Kindness Meditation.* Dissertations & Theses, Gradworks Ltd, Coleraine.

[B59] Xu W. Wang Y. Liu X. (2015). Effectiveness of 8-week mindfulness training improving negative emotions. *Chin. Ment. Health J.* 29 497–502. 10.3969/j.issn.1000-6729.2015.07.004

[B60] Yang G. (2011). The Topic-based Workshop Mode in Accomplished Teachers’. *Train. J. Zhejiang Edeuc. Inst.* 1 8–12. 10.3969/j.issn.2095-2074.2011.01.002

[B61] Yang T. Z. Huang H. T. (2003). An epidemiological study on stress among urban residents in social transition period. *Chin. J. Epidemiol.* 24:760. 14521764

[B62] Yi X. Zhao Q. Hu W. (2014). The cross-temporal analysis of mental health of Chinese teachers:1994 2011. *J. Beijing Norm. Univ. Soc. Sci.* 3 13–23.

[B63] Zeidan F. Gordon N. S. Merchant J. Goolkasian P. (2010a). The effects of brief mindfulness meditation training on experimentally induced pain. *J. Pain.* 11 199–209. 10.1016/j.jpain.2009.07.015 19853530

[B64] Zeidan F. Johnson S. K. Gordon N. S. Goolkasian P. (2010b). Effects of brief and sham mindfulness meditation on mood and cardiovascular variables. *J. Altern. Complement. Med.* 16 867–873. 10.1089/acm.2009.0321 20666590

[B65] Zembylas M. Schutz P. A. (2009). “Research on teachers’ emotions in education: findings, practical implications and future agenda,” in *Advances in Teacher Emotion Research*, eds Schutz P. A. Zembylas M. (New York: NY: Springer US).

