# Supplementary material for: Effects of a Four-Day Mindfulness Intervention on Teachers’ Stress and Affect: A Pilot Study in Eastern China
Source: Front Psychol. 2020 Jun 30;11:1298. doi: 10.3389/fpsyg.2020.01298 (PMC7338718; doi:10.3389/fpsyg.2020.01298)
Supplement: Supplementary file 1 [file Data_Sheet_1.docx]

***Supplementary Material***

**1. Supplementary Table 1**

***Course schedule (core practice)***

|  | Day 1 | Day 2 | Day 3 | Day 4 |
| --- | --- | --- | --- | --- |
| 9:00-12:00a.m. | A short introduction of mindfulness | Mindful Hatha Yoga | a whole day silent retreat, including sitting meditation, mindful stretch /walking, body scan, Hatha Yoga, mindful eating and resting, and ended with a compassion meditation; | Mindful walking |
|  | Raisin practice | Sitting meditation(30mins) |  | Choiceless awareness meditation |
|  | Sitting meditation and mindful awareness of breath | The pleasant event practice |  | Mindful Tai Chi |
|  | | |  |  |
| 2：00-5:00p.m. | Body scan | Body scan |  | Body scan |
|  | Mindful walking | The unpleasant event practice |  | A discussion about bringing mindfulness into real life |
|  | Lecture about mindfulness training (60 mins) | The mountain meditation |  | Sharing within group |
|  |  |  |  | Ending in a compassion meditation |
