# Supplementary material for: Effects of a Four-Day Mindfulness Intervention on Teachers’ Stress and Affect: A Pilot Study in Eastern China
Source: Front Psychol. 2020 Jun 30;11:1298. doi: 10.3389/fpsyg.2020.01298 (PMC7338718; doi:10.3389/fpsyg.2020.01298)
Supplement: Supplementary file 2 [file Data_Sheet_2.docx]

***Supplementary Material***

**2. Supplementary Material 2:**

**Mindfulness intervention improves emotion and decreases stress through increasing mindfulness**

Former studies have shown that trait mindfulness predicts less stress (Bulli et al., 2013). Roeser et al. (2013) tested whether the mindfulness level at post-program mediated the effects of mindfulness training in reducing stress and burnout at a three-month follow-up. Furthermore, Bergman et al. (2016) found that changes in mindfulness predict stress and anger outcomes in police officers. Therefore, we conducted an additional mediational analysis. We hypothesized that mindfulness level would improve after the intervention and tested a model to determine whether the change of outcome was brought by a change in the level of mindfulness. We speculated that a change in the level of mindfulness could mediate the effect of the intervention on the outcomes. The mediation model was tested. We explored the relationship between increase in mindfulness and mental health by calculating the correlations between percent change in all variables.

As illustrated in table, the degrees of change for most variables were significantly correlated, except for positive affect and mindfulness. It is worth noting that the degree of change in mindfulness was correlated with negative affect and stress. (See Supplementary Table 2).

We modified the model and tested the hypothesis that the degree of change in mindfulness would mediate the relationship between mindfulness training and mental health outcomes. Due to the non-significance between the percent change in mindfulness and the percentage change in positive affect, only two models were tested:

Groups 🡒 Percent change in mindfulness 🡒 Percent change in negative affect

Groups 🡒 Percent change in mindfulness🡒 Percentage change in stress

*^*^Groups indicate mindfulness and waitlist groups*

In order to understand the change more accurately, the percentage change in outcome was analyzed in this model. We assigned dummy variables to both groups: 1 for the mindfulness group and 0 for the control group. We then conducted a mediational analysis of 5,000 bootstrap resamples. Figure 3 shows the results; linear regression showed that the intervention could significantly predict the percent change in the MAAS score ($\beta$*=*11.32^**^*^*^,* 95%*CI* [4.73, 17.72]). For negative affect, the direct effect of the intervention on the percent change in outcome was significant ($\beta=-$9.60*^*^*, 95%*CI* [-18.38, -.41]), and when the effect of mindfulness was added to the model ($\beta=-.46$ *^***^*, *95%CI* [-.66, -.25]), the coefficient of the indirect effect of the intervention on negative affect was not significant ($\beta=-4.41$, 95%*CI* [-13.05, 4.57]). For stress, the intervention could also significantly predict percent change in stress ($\beta=-5.85$*^*^*, 95%*CI* [-11.49, -.19])*.* After adding the effect of mindfulness ($\beta=-.36$*^**^*^*^,95%*CI* [-.49, -.24])*,* the coefficient of the indirect effect of the intervention on stress was not significant$(\beta=-1.74$*,* 95%*CI* [-7.10, 3.66])*.* The results suggest that the mindfulness intervention does decrease negative affect and stress by increasing the level of mindfulness (See Supplementary Figure 1).

| **Supplementary Table 2**  **Correlations Between Change Values in the Mindfulness Group** | | | | |
| --- | --- | --- | --- | --- |
|  | %change of MAAS | %change of PA | %change of NA | %change of STRESS |
| %change of MAAS | - |  |  |  |
| %change of PA | .13 [-.094, .35] ^a^ | - |  |  |
| %change of NA | -.31^**^[-.50, -.090] | -.29^*^[-.48, -.068] | - |  |
| %change of STRESS | -.36^***^[-.54, -.14] | -.42^***^[-.59, -.22] | .55^*^[.37 .69] | - |
| Note.  T1=baseline; T2=post-program; %change= percentage change=$\frac{T2-T1}{T1}*100\%$  ^a^ Pearson’s r; [95% Confidence Interval]  ^*^ p<.05, ^**^p<.01, ^***^p<.001 | | | | |


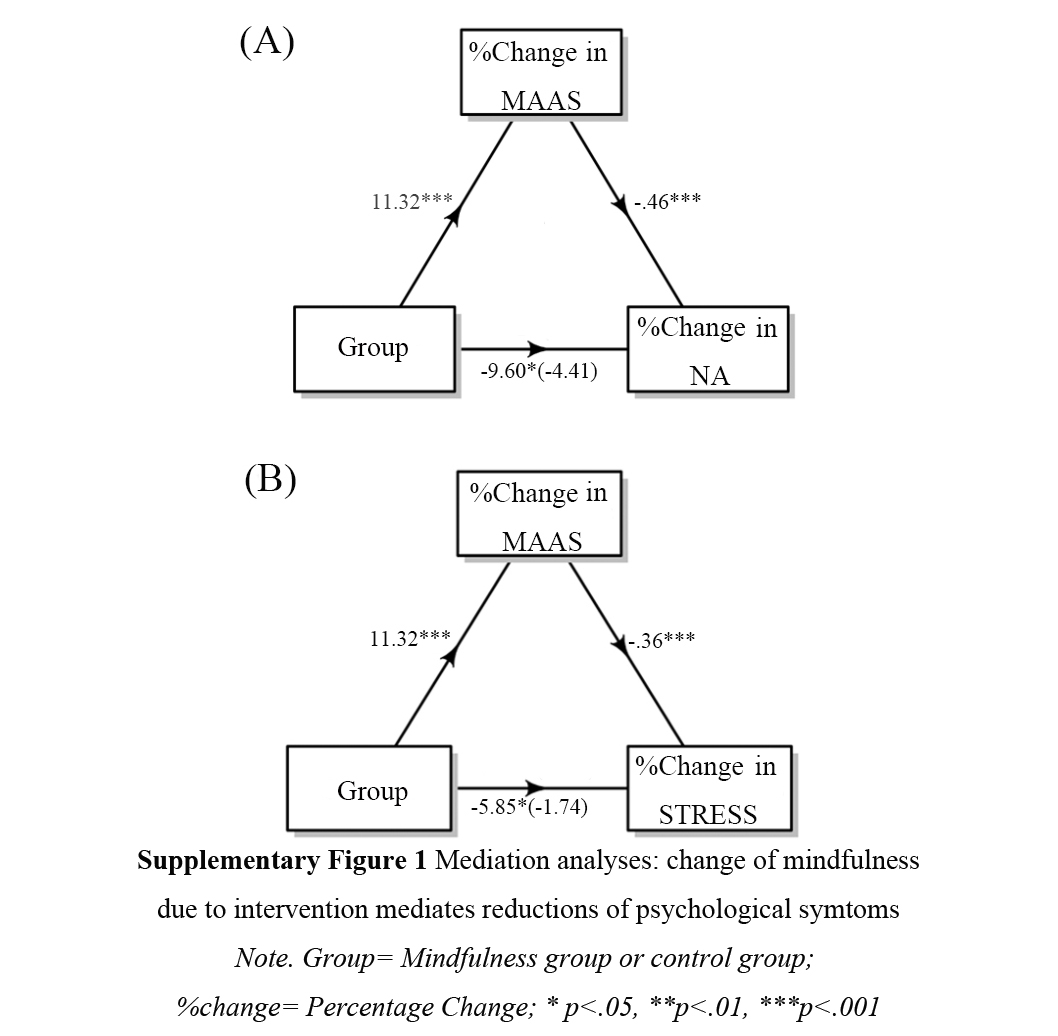


The complete mediation model explained the route between mindfulness training and emotional health. That is, improvements in the mindfulness group’s negative affect and stress were explained by improvements in their mindfulness. Combined with the significant increase in their mindfulness, the complete mediation model indicated that paying attention to the present could significantly help transform negative emotions and perceived psychological stress. More importantly, this capacity can be developed and strengthened by intensive mindfulness practices over several days.

**Reference:**

Bergman, A. L., Christopher, M. S., and Bowen, S. (2016). Changes in facets of mindfulness predict stress and anger outcomes for police officers. *Mindfulness*. *7*(4), 851–858. https://doi.org/10.1016/j.jbtep.2013.07.006

Bullis, J. R., Bøe, H. J., Asnaani, A., and Hofmann, S. G. (2013). The benefits of being mindful: trait mindfulness predicts less stress reactivity to suppression. *J Behav Ther Exp Psychiatry*. *45*(1), 57–66. https://doi.org/10.1016/j.jbtep.2013.07.006

Roeser, Robert W., Schonert-Reichl, K. A., Jha, A., Cullen, M., Wallace, L., Wilensky, R., … Harrison, J. (2013). Mindfulness training and reductions in teacher stress and burnout: results from two randomized, waitlist-control field trials. *J. Edu. Psychol.* *105*(3), 787–804. https://doi.org/10.1037/a0032093
